# A Framework to Examine the Role of Epigenetics in Health Disparities among Native Americans

**DOI:** 10.1155/2013/410395

**Published:** 2013-12-09

**Authors:** Teresa N. Brockie, Morgan Heinzelmann, Jessica Gill

**Affiliations:** Nursing Research and Translational Science, National Institutes of Health, Clinical Center, 10 Center Drive, Room 2B11, Bethesda, MD 20852, USA

## Abstract

*Background*. Native Americans disproportionately experience adverse childhood experiences (ACEs) as well as health disparities, including high rates of posttraumatic stress, depression, and substance abuse. Many ACEs have been linked to methylation changes in genes that regulate the stress response, suggesting that these molecular changes may underlie the risk for psychiatric disorders related to ACEs. *Methods*. We reviewed published studies to provide evidence that ACE-related methylation changes contribute to health disparities in Native Americans. This framework may be adapted to understand how ACEs may result in health disparities in other racial/ethnic groups. *Findings*. Here we provide evidence that links ACEs to methylation differences in genes that regulate the stress response. Psychiatric disorders are also associated with methylation differences in endocrine, immune, and neurotransmitter genes that serve to regulate the stress response and are linked to psychiatric symptoms and medical morbidity. We provide evidence linking ACEs to these epigenetic modifications, suggesting that ACEs contribute to the vulnerability for developing psychiatric disorders in Native Americans. *Conclusion*. Additional studies are needed to better understand how ACEs contribute to health and well-being. These studies may inform future interventions to address these serious risks and promote the health and well-being of Native Americans.

## 1. Introduction

**Figure 1:**
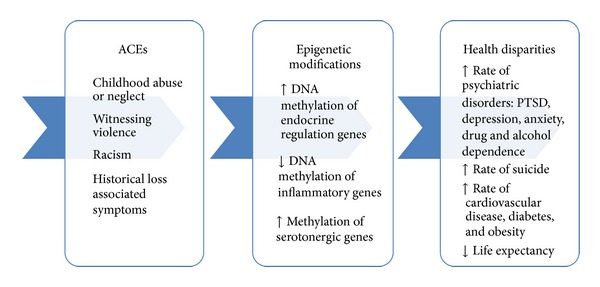
The mediating relationship of epigenetics on the risk for health disparities in Native Americans with childhood adversity.

Reservation-based Native Americans live in pervasively adverse social and physical environments that place them at increased risk of exposure to a myriad of stressors during childhood which impact their psychological and physical health over their lifetimes [1]. About 1 of 2.9 million Native Americans that identify as Native American alone resides on reservations [2]. Indian reservations were established by treaty during the Removal and Relocation (1827–1887) period and are lands set aside for tribes in exchange for ceded land and resources. Today there exist 275 Indian land areas in the USA administered as Indian reservations [3]. Of the ten poorest counties in America, five are home to an Indian reservation [4]. Concentrated poverty results in higher crime rates, underperforming public schools, poor housing, and poor health and limits access to many services and job opportunities [5]. Adverse childhood experiences (ACEs) that are substantial contributors to health disparities include childhood physical and sexual abuse, witnessing violence, poverty, and racism. The concept that these experiences become biologically embedded has gained substantial support and provides an explanatory mechanism for health disparities [6]. ACEs are linked to differences in the function of the stress-response system including the neuroendocrine system, the parasympathetic nervous system, and the immune system. These changes likely have substantial long and short term impacts on health and well-being [7]. It is likely that these changes are shaped by epigenetic modifications which alter the function but not the structure of the gene. Epigenetic modifications are considered to be an individual's molecular response to the environment and occur in an effort to preserve the health of the individual by increasing the accessibility of genes for transcription and translation that relate to immediate survival [8]. These genes code for proteins that prepare the individual to be able to respond to the stressor through a fight or flight response; yet, in Native Americans living on reservations, the stressors most encountered are chronic, not acute. Thus, this adaptive response likely results in overactivation of this stress-response system, and this excessive activity has substantial negative consequences on the health and well-being of Native Americans, individually and across generations. Here we provide a conceptual review of how nurses and other health care professionals can examine health disparities in Native Americans through epigenetic modifications that likely result from ACEs (see Figure 1), including historical trauma, the residual of which is assumed to be historical loss associated symptoms. We expect this conceptual framework to have implications for or be relevant to the mechanisms of health disparities in other racial or ethnic groups.

## 2. ACEs and Psychiatric Risks in Native Americans

A neighborhood's safety and access to quality health care, economic opportunities, social connections, and social capital are all key determinants of the health of its residents over time [9–12]. Reservations are often characterized by low economic status and segregation, both of which limit access and are risk factors for higher rates of morbidity and mortality [13, 14]. Chronic stress such as that which accompanies experiences of racism and poverty over a lifetime places individuals at risk for posttraumatic stress disorder (PTSD). This vulnerability can be solved in part by ethnic connectedness [15].

Unique to Native Americans is the race-based stress associated with historical trauma [16], as well as discrimination [17–20]. Historical trauma is defined here as the “collective experience of violence perpetrated against Indigenous Peoples in the process of colonizing the Americas resulting in an unresolved humanitarian crisis for reservation communities.” The effects of historical trauma are proposed as being transmitted across generations with historical loss associated symptoms currently exhibited [21–23] and include symptoms of complicated bereavement and complex PTSD [24]. This type of trauma has been linked to impaired individual and collective tribal identity [16, 24], which likely also relates to stress and morbidity risk. Over 50% of Native Americans indicate that they think about loss related to historical trauma, such as loss of language, loss of culture, and loss of land, at least occasionally, and which caused them psychological distress [17, 25]. Discrimination has been associated with early substance use among Native American children, and suicidal behavior, and anger, and aggression among adolescents [18, 20, 26]. Thus, this stress combined with other ACEs may be a significant contributor to health disparities.

Native Americans are disproportionally affected by trauma in childhood, including abuse, neglect, and exposure to intimate partner violence (IPV) [27–29]. Approximately half of Native American adolescents and young adults have been exposed to one or more severe traumatic events [30], and 98% have experienced a traumatic event of any severity [31]. Native American adolescents are more likely than other adolescents to witness violence or to have been physically abused, sexually abused, or neglected as a child, resulting in rates of PTSD that are twice that of the estimated rates in the general U.S. population [31].

Assaultive trauma in childhood is linked to the highest risk for PTSD, suggesting that this ACE is specifically linked to this high risk for psychiatric disorders [31]. Specifically, trauma that involves physical or sexual assault prior to adolescence places an individual at five to ten times the risk for PTSD onset compared to an individual without this experience [32, 33]. Witnessing abuse during childhood, as well as residing in a high-crime area, is also linked to a far greater risk for PTSD [34, 35]; however, assaultive trauma at an early age is the ACE most linked to PTSD onset.

ACEs have also been linked to increased risk of depression onset [36–38]. These studies link physical abuse, witnessing domestic violence, and parental alcohol and drug abuse to a vulnerability for depression symptom onset [36, 37]. In addition, residing in an urban, socioeconomically disadvantaged area has also been linked to risk of depression onset as well as drug use [39]. Exposure to trauma also increases the risk for the early onset of substance use and the onset of substance use disorder [40]. Other studies have found similar results, including that ACEs increase the risk for drug use and early alcohol abuse and increased the rates of initiating these behaviors during adolescence by a factor of two to four [41, 42]. Thus, ACES in general are linked to psychiatric disorder vulnerability, with high degree of comorbidity among these disorders.

## 3. Health Disparities in Native Americans

Reservation-based Native Americans die at higher rates than other Americans from tuberculosis (750% higher), alcoholism (524% higher), diabetes (293% higher), unintentional injuries (153% higher), homicide (103.3% higher), and suicide (66% higher) (2002–2004, rates adjusted for misreporting of race on state death certificates) [43]. Not only do Native Americans bear a disproportionate burden of disease, but they also experience a lower life expectancy. Life expectancy is an overall measure of quality of life [44] and is one of the indicators used to measure the magnitude of the burden of health disparities [45]. In general, Native Americans born in 2000–2002 have a life expectancy that is about 2.4 years less than the overall US population rate: 76.9 years compared to 74.5 years for Native Americans [43]. However, when this average is disaggregated by IHS Area, the life expectancy ranges from 64.8 years (11 years less than for the U.S.) in the Aberdeen Area to 76.4 years (greater than the U.S. average of 75.8 years) in the California Area (adjusted for race miscoding) using 1994–1996 data [46]; thus highlighting the within group differences. Additionally, the Indian Health Service, using 2000 census data, found 25.7% of all Native Americans were living below the poverty level, compared to 12.4% of the U.S. population overall [43]. The Bureau of Justice, in the first comprehensive statistical analysis of “American Indians and Crime,” reports Native American are the victims of violent crimes at two times the rate of the U.S. population overall, and about 7 in 10 violent victimizations involved an offender who was reported by the victim to be a person of another race [47]. However, this may not apply to all communities, especially those that are more remote and isolated where few non-Native American people live. Another report by the Department of Justice, disclosed Native Americans sustain rates of violent victimization (rape, sexual assault, robbery, aggravated assault, and simple assault) at rates that are 2 times higher than African Americans, 2.5 times that of Hispanics, 3 times that of Caucasians, and 6.5 times that of Asians [48]. PTSD is the anxiety disorder most linked to trauma and its prevalence in Native Americans adults is 4.4 times the national average [25, 49]. There is little research regarding the impact that adversity has on tribal communities, so it remains poorly understood.

The adverse childhood experiences (ACE) study suggests that certain adversities are major risk factors for morbidity and mortality [50]. The study established a relationship between adversity in childhood and suicide attempt [51], prescription drug use [52], alcoholism and alcohol abuse [53, 54], illicit drug use [42], obesity [55], and depressive disorders [38]. Among adolescents and young adults, childhood adversity was also associated with a greater risk for interpersonal violence perpetration [56], poor perceived health, more medical care visits, and additional somatic concerns [57]. Therefore, current studies link ACEs to risks to health and well-being; however, the mechanisms underlying these risks have not yet been well described.

## 4. Genetic Inheritance and Influences on ACEs and Health

In some cases, genetic predisposition may explain some of the enduring effects of ACEs; however, the evidence for this link remains poorly understood. Genetic inheritance provides information encoded in DNA which is transcribed to various types of RNA molecules which likely shape the response of the individual to stressors such as ACEs. One important concept related to phenotypic variation is heritability, which estimates the extent of which genetic inheritance contributes to the phenotypic variance in a population [58]. Heritability is the percent of variation in the genome responsible for the difference in the phenotype. Another parameter used to estimate the contribution of genomic factors in phenotypes is relative risk, which refers to an individual's risk of developing a condition with a family history compared to those without a history [58]. When the heritability estimate or relative risk of a phenotype is low, the influence of the genome sequence is considered to be relatively smaller than the influence of other factors such as environment, and the genomic influence can be easily masked or have a negligible impact. Since most human diseases involve many genes, their interactions, and nongenetic factors, an approach termed “endophenotypes” is used to characterize the disease in a molecular or genetic manner, rather than using a clinical diagnosis to define the phenotype.

Polymorphisms in Native Americans have been linked to a greater vulnerability for alcohol abuse [59], as well as obesity [60]. In general, U.S. samples of trauma exposed participants link endocrine gene (FKBP5) polymorphisms to a greater risk for PTSD development [59, 61]; yet, these are small and do not include Native Americans. Thus, it is essential to consider unique genetic inheritance features in Native Americans which interact with epigenetic modifications and likely contribute to health disparities.

### 4.1. ACEs and the Biological Stress Response

The stress-response system provides the individual protection from acute stressors through an activation of interactive biological systems [6]. One biological system that is central to this response and is linked to ACEs is the hypothalamic-pituitary-adrenal (HPA) axis, with the end result of activation of this system being the production of cortisol. In addition to playing a pivotal role in activating the stress response, the HPA axis also influences biological functions related to mood, growth, immune function, metabolism, and regulation of biological systems on circadian rhythm [62]. The sympathetic nervous system (SNS) also is activated by stress providing neuronal focus and energy to muscles in order to escape the stressor. Although these systems are effective in adapting to acute stress, chronic activation is linked to negative consequences. Both the HPA axis and SNS impact immune function, and chronic stress is linked to a risk for inflammation [62]. Overactivation of the HPA axis results in disruptions of functioning at rest and following stressors, and these changes have been linked to ACEs. HPA axis alterations are linked to health disparities through mechanisms that include impaired neuronal growth and survival, inflammation, reductions in neuropeptide activity, and accelerated cellular aging [63–65]. SNS function changes have also been linked to health disparities, with one of the most pivotal mechanisms being a lack of circadian variation in blood pressure, a key risk factor for myocardial infarctions [66].

## 5. Epigenetic Modifications Resulting from ACEs

Evidence is accumulating that environmental influences early in development remain pervasive into adulthood, a relationship that is attributed to an interaction of gene function and environment. Both genetic and environmental factors are critical to developmental processes and even minor changes in either type of factor can result in trajectories of resilience or vulnerability [67]; however, it is the interaction between these factors that may provide the most vital information to understand the heterogeneous response to trauma. This leads us and others to question how future research can address this critical issue.

Epigenetics refers to changes in an individual's phenotype independent of genotype. These changes occur through mechanisms such as histone modification, methylation, acetylation, and noncoding ribonucleic acids which alter the accessibility of genes for transcription. The resulting transcription modifications and protein production result from factors such as environmental challenges including, but not limited to, ACEs [68, 69]. An individual's genome interacts with internal and external factors to create phenotypes such as height, physical appearance, personality, and alterations in the stress-response system [70].

Preclinical models illustrate how ACEs result in epigenetic modifications in neurons, thereby increasing the risk for psychiatric symptoms. To illustrate this link, a study reports that the offspring of high-licking canine mothers exhibit reduced methylation of the glucocorticoid receptor gene [71] and endocrine regulation of a subsequent stressor [72]. In contrast, offspring that face early adversity exhibit endocrine dysregulation [73], as well as reductions in neuronal plasticity in the prefrontal cortex (PFC) that persist into adulthood [74]. In studies of rats who exhibit PTSD-like behavior, there is evidence of increased methylation of stress-response genes including brain-derived neurotrophic factor and nuclear protein phosphate-1 [75] in neurons [76]. Although these studies provide additional evidence linking ACEs to methylation changes in neurons, these studies are not able to clearly determine psychiatric symptoms. Therefore, these studies are limited by not being able to determine the comprehensive risks that relate to ACEs.

The ACE most linked to epigenetic differences and vulnerability for health disparities is that of child abuse. To illustrate this link, in a hallmark study by Labonté in suicide completers, ACEs were linked to increased DNA methylation of the glucocorticoid receptor in the hippocampus, and this differential methylation was particularly linked to childhood abuse [77]. This study provides further support for the McGowan et al. study, whose subject group was also suicide completers, which reported that childhood abuse was associated with greater methylation levels at CpG sites in the exon1_F_ of the promoter region of the glucocorticoid receptor gene [78]. These studies had the distinct advantage of examining epigenetic modifications in neurons, which is not available in other studies. Epigenetic patterns differ among cell types, even differing among brain regions [79, 80]. Thus, an additional challenge to understanding the impact of ACEs on health disparities is to determine how epigenetic alterations in the brain differ from those in peripheral tissues and how to advance despite this methodological challenge. In addition, these few studies are not able to determine the role of preexisting methylation in this risk or to measure other factors that may contribute to methylation changes.

Epigenetic changes resulting from ACEs can also be observed in studies that use peripheral blood in living participants, which show that HPA-regulating genes are often impacted. A study by Klengel et al. linked ACEs to reduced methylation of the FKBP5 gene, an essential regulator of the stress response, as well as to changes in the function of the HPA axis under stress, and to reduced cognitive ability [81]. Direct physical abuse and observing the abuse of a mother have also been associated with greater methylation levels at CpG sites in the exon1_F_ of the promoter region of the glucocorticoid receptor gene in leukocytes [82]. Similar methylation profiles are also reported in the peripheral blood of babies whose mothers were depressed during the third trimester of pregnancy, and these methylation changes were related to salivary cortisol elevations at three months of age [83]. Although glucocorticoid receptors in peripheral tissue may differ from those on the HPA axis, the link between methylation of the glucocorticoid gene in the periphery and the function of the HPA axis has been demonstrated in multiple studies in addition to those of Oberlander et al., 2008. Additional studies that include analysis of blood samples collected closer to the time of the ACE may provide additional insights into the individual variation in response to ACEs.

Other studies link ACEs to hypomethylation of inflammatory genes, suggesting that these experiences result in a greater inflammation later in life. In a recent study of children who were removed from their parents due to abuse or neglect, a reduction in methylation of NR3C1, an inflammatory regulation gene, as well as differential methylation of cancer related pathways was found in children with ACEs compared to controls [84]. A study of adults linked child abuse to reduced methylation of IGF2AS, an antisense transcript of the insulin-like growth factor gene, which encodes for the inflammatory cytokine family of growth factor beta [85]. Borghol et al. linked childhood poverty to differential methylation of genes related to metabolism and inflammation, and these changes were different from those in participants who experience poverty only during adulthood [86]. Together these studies provide evidence that a variety of ACEs result in methylation changes, suggesting that these molecular changes likely contribute to health disparities; however, additional, larger, and more representative studies are needed to determine relationships.

Altered serotonergic neurotransmission is also postulated to result from ACEs and provides a mechanistic link to increased vulnerability for psychiatric disorders. The Iowa adoption study demonstrated a link between hypermethylation of the serotonin gene SLC64A to childhood sex abuse [87], and this molecular change mediated the development of antisocial personality disorder [88]. This group was also able to relate differences in gene expression of serotonin related genes to methylation, and that genotype influenced methylation at cg22584138 [89]. Additional studies are needed to determine the role of other ACEs in serotonergic gene methylation and to determine how ACEs contribute to psychiatric risks.

## 6. Methylation Changes Associated with ACEs Increase the Risk for Psychiatric Disorder Onset

Clinical studies are restricted to examining differential methylation in samples of peripheral fluids, but these studies do provide some key insights into how these molecular changes relate to PTSD, depression, and drug abuse risk. For instance, PTSD is associated with changes in the methylation of inflammatory (toll-like receptors 1 & 3, IL-8, chemokine ligand 1, and others) and endocrine genes FK506 binding protein-5 (FKBP5) [90]. Another study that measured DNA methylation reported that postdeployment hypomethylation of LINE-1 was associated with PTSD onset following deployment [91]. Differential methylation of neurotransmitter genes is also linked to PTSD risk. Two studies utilized samples of civilians from the Detroit Neighborhood Health Study. One study determined that serotonin transporter gene (SLC6A4) methylation levels were modified by the effect of the number of traumatic events on PTSD after controlling for SLC6A4 genotype, such that persons with more traumatic events were at increased risk for PTSD, but only at lower methylation levels [92]. The other study found that the candidate gene MAN2C1 showed a significant methylation × trauma experience interaction, such that those with both higher MAN2C1 methylation and greater exposure to traumatic events showed an increase in risk of lifetime PTSD [93]. Thus, there is evidence that PTSD is associated with similar methylation differences in immune, endocrine, and neurotransmitter genes to those linked to ACEs; suggesting that these chronic differences may be a result of ACEs, yet additional prospective studies are needed to better describe these relationships.

Studies in individuals with depression have also shown differential DNA methylation. Sabunciyan et al. carried out the first genome-wide DNA methylation scan in major depressive disorder patients. The study pinpointed 224 candidate regions, primarily involved in neuronal growth and development genes, which showed differential methylation; PRIMA1 showed the greatest differences [94]. Specific genes that have been shown to be differentially methylated in individuals with depression include those that code for angiotensin converting enzyme [95], brain-derived neurotrophic factor [96], orexin A [97], and gamma-aminobutyric acid receptor alpha1 [98].

In addition to observing differential DNA methylation in PTSD and depression, many studies have observed methylation differences in those that suffer from drug abuse as compared to healthy controls. Increases in DNA methylation of the OPRM1 gene that codes for opioid receptors have been reported in individuals with chronic opioid use [99–102] and alcohol dependence [103], and global methylation differences have also been reported for these two populations [99, 104]. The proopiomelanocortin gene promoter [105], dopamine transporter gene promoter [106], homocysteine-induced endoplasmic reticulum protein promoter [107], and alpha synuclein promoter [108] were found to be differentially methylated in individuals with alcoholism compared to healthy controls. Methylation at the monoamine oxidate A locus was also significantly associated with nicotine and alcohol dependence in women, but not in men [109]. Together these studies show that psychiatric disorders related to ACEs are associated with methylation changes that may be reflective of ACEs or psychiatric symptoms; however, there are no prospective studies to elucidate the possible mediating role of methylation on these psychiatric risks in individuals that experience ACEs.

## 7. Conclusion

Reservation-based Native Americans disproportionately experience ACEs and health disparities, significantly impacting long-term physical and psychological health. In addition to these experiences, the persistence of stress associated with discrimination and historical trauma converges to add immeasurably to these challenges. Here we provide evidence to suggest that ACEs result in methylation differences in genes that regulate the stress response and that these changes may contribute to an increased vulnerability for developing psychiatric disorders, as depicted in Figure 1. Although we postulate these relationships, the lack of prospective studies in this at-risk group prevents us and others from concluding this causality, as well as more studies that include Native Americans. Thus, additional studies are needed to better understand the mechanisms through which ACEs contribute to health and well-being. These studies may inform future interventions to address these serious risks and promote the health and well-being of Native Americans.

## References

[1] Willows ND, Hanley AJG, Delormier T (2012). A socioecological framework to understand weight-related issues in Aboriginal children in Canada. *Applied Physiology, Nutrition and Metabolism*.

[2] Cornell S, Kalt JP (2010). American Indian self-determination: the political economy of a successful policy. *Joint Occasional Papers on Native Affairs*.

[3] Bureau of Indian Affairs (2011). *Frequently Asked Questions*.

[4] MainStreet (2011). *The Poorest Counties in America*.

[5] The Brookings Institute Concentrated poverty. http://www.brookings.edu/research/topics/u-s-poverty.

[6] Ehlert U (2013). Enduring psychobiological effects of childhood adversity. *Psychoneuroendocrinology*.

[7] Hostinar CE, Sullivan RM, Gunnar MR (2013). Psychobiological mechanisms underlying the social buffering of the hypothalamic-pituitary-adrenocortical axis: a review of animal models and human studies across development. *Psychological Bulletin*.

[8] Kuzawa CW, Sweet E (2009). Epigenetics and the embodiment of race: developmental origins of US racial disparities in cardiovascular health. *American Journal of Human Biology*.

[9] Diez Roux AV (2003). Residential environments and cardiovascular risk. *Journal of Urban Health*.

[10] Diez Roux AV, Jacobs DR, Kiefe CI (2002). Neighborhood characteristics and components of the insulin resistance syndrome in young adults: the Coronary Artery Risk Development in Young Adults (CARDIA) study. *Diabetes Care*.

[11] Whitaker D, Milam AJ, Graham CM, Cooley-Strickland M, Belcher HM, Furr-Holden CD (2013). Neighborhood environment and urban schoolchildren's risk for being overweight. *American Journal of Health Promotion*.

[12] O’Campo P, Xue X, Wang M-C, Brien Caughy MO (1997). Neighborhood risk factors for low birthweight in baltimore: a multilevel analysis. *American Journal of Public Health*.

[13] Chaix B, Jouven X, Thomas F (2011). Why socially deprived populations have a faster resting heart rate: impact of behaviour, life course anthropometry, and biology—the RECORD Cohort Study. *Social Science and Medicine*.

[14] Lawman HG, Wilson DK (2012). A review of family and environmental correlates of health behaviors in high-risk youth. *Obesity*.

[15] Khaylis A, Waelde L, Bruce E (2007). The role of ethnic identity in the relationship of race-related stress to PTSD symptoms among young adults. *Journal of Trauma and Dissociation*.

[16] Brave Heart MY, DeBruyn LM (1998). The American Indian Holocaust: healing historical unresolved grief. *American Indian and Alaska Native Mental Health Research*.

[17] Whitbeck LB, Chen X, Hoyt DR, Adams GW (2004). Discrimination, historical loss and enculturation: culturally specific risk and resiliency factors for alcohol abuse among American Indians. *Journal of Studies on Alcohol*.

[18] Whitbeck LB, Hoyt DR, McMorris BJ, Chen X, Stubben JD (2001). Perceived discrimination and early substance abuse among American Indian children. *Journal of Health and Social Behavior*.

[19] Whitbeck LB, McMorris BJ, Hoyt DR, Stubben JD, LaFromboise T (2002). Perceived discrimination, traditional practices, and depressive symptoms among American Indians in the upper midwest. *Journal of Health and Social Behavior*.

[20] Freedenthal S, Stiffman AR (2004). Suicidal behavior in urban American Indian adolescents: a comparison with reservation youth in a southwestern state. *Suicide and Life-Threatening Behavior*.

[21] Yehuda R, Schmeidler J, Giller EL, Siever LJ, Binder-Brynes K (1998). Relationship between posttraumatic stress disorder characteristics of holocaust survivors and their adult offspring. *The American Journal of Psychiatry*.

[22] Yehuda R, Schmeidler J, Wainberg M, Binder-Brynes K, Duvdevani T (1998). Vulnerability to posttraumatic stress disorder in adult offspring of Holocaust survivors. *The American Journal of Psychiatry*.

[23] Brave Heart MYH (1996). The return to the sacred path: healing from historical trauma and historical unresolved grief among the Lakota. *Dissertation Abstracts International A*.

[24] Gone JP (2009). A community-based treatment for native American historical trauma: prospects for evidence-based practice. *Journal of Consulting and Clinical Psychology*.

[25] Ehlers CL, Gizer IR, Gilder DA, Yehuda R (2013). Lifetime history of traumatic events in an American Indian community sample: heritability and relation to substance dependence, affective disorder, conduct disorder and PTSD. *Journal of Psychiatric Research*.

[26] Sittner Hartshorn KJ, Whitbeck LB, Hoyt DR (2012). Exploring the relationships of perceived discrimination, anger, and aggression among North American indigenous adolescents. *Society and Mental Health*.

[27] Robin RW, Chester B, Rasmussen JK (1998). Intimate violence in a Southwestern American Indian tribal community. *Cultural Diversity and Mental Health*.

[28] Manson SM, Beals J, Klein SA, Croy CD (2005). Social epidemiology of trauma among 2 American Indian reservation populations. *American Journal of Public Health*.

[29] Wahab S, Olson L (2004). Intimate partner violence and sexual assault in Native American communities. *Trauma, Violence & Abuse*.

[30] Boyd-Ball AJ, Manson SM, Noonan C, Beals J (2006). Traumatic events and alcohol use disorders among American Indian adolescents and young adults. *Journal of Traumatic Stress*.

[31] Deters PB, Novins DK, Fickenscher A, Beals J (2006). Trauma and posttraumatic stress disorder symptomatology: patterns among american indian adolescents in substance abuse treatment. *American Journal of Orthopsychiatry*.

[32] Breslau N (2002). Psychiatric morbidity in adult survivors of childhood trauma. *Seminars in Clinical Neuropsychiatry*.

[33] Bromet E, Sonnega A, Kessler RC (1998). Risk factors for DSM-III-R posttraumatic stress disorder: findings from the national comorbidity survey. *American Journal of Epidemiology*.

[34] Stevens NR, Gerhart J, Goldsmith RE, Heath NM, Chesney SA, Hobfoll SE (2013). Emotion regulation difficulties, low social support, and interpersonal violence mediate the link between childhood abuse and posttraumatic stress symptoms. *Behavior Therapy*.

[35] Sloan-Power EM, Boxer P, McGuirl C, Church R (2013). Coping zone construction and mapping: an exploratory study of contextual coping, PTSD, and childhood violence exposure in urban areas. *Journal of Interpersonal Violence*.

[36] Sareen J, Henriksen CA, Bolton SL, Afifi TO, Stein MB, Asmundson GJ (2013). Adverse childhood experiences in relation to mood and anxiety disorders in a population-based sample of active military personnel. *Psychological Medicine*.

[37] de Venter M, Demyttenaere K, Bruffaerts R (2013). The relationship between adverse childhood experiences and mental health in adulthood. A systematic literature review. *Tijdschrift Voor Psychiatrie*.

[38] Chapman DP, Whitfield CL, Felitti VJ, Dube SR, Edwards VJ, Anda RF (2004). Adverse childhood experiences and the risk of depressive disorders in adulthood. *Journal of Affective Disorders*.

[39] Schilling EA, Aseltine RH, Gore S (2007). Adverse childhood experiences and mental health in young adults: a longitudinal survey. *BMC Public Health*.

[40] Whitesell NR, Beals J, Mitchell CM, Manson SM, Turner RJ (2009). Childhood exposure to adversity and risk of substance-use disorder in two American Indian populations: the meditational role of early substance-use initiation. *Journal of Studies on Alcohol and Drugs*.

[41] Anda RF, Felitti VJ, Bremner JD (2006). The enduring effects of abuse and related adverse experiences in childhood: a convergence of evidence from neurobiology and epidemiology. *European Archives of Psychiatry and Clinical Neuroscience*.

[42] Dube SR, Felitti VJ, Dong M, Chapman DP, Giles WH, Anda RF (2003). Childhood abuse, neglect, and household dysfunction and the risk of illicit drug use: the adverse childhood experiences study. *Pediatrics*.

[43] Indian Health Service (2009). *Trends in Indian Health 2002-2003 Edition*.

[44] Williams RA (2007). *Eliminating Healthcare Disparities in America: Beyond the IOM Report*.

[45] Smedley BD, Stith AY (2003). *Unequal Treatment: Confronting Racial and Ethnic Disparities in Health Care*.

[46] Indian Health Service (2003). *Regional Differences in Indian Health 2000-01*.

[47] Greenfeld L, Smith SK (1999). American Indians and crime. *ABJS Statistical Profile, 1992-2002*.

[48] Truman JL (2011). *Criminal Victimization, 2012*.

[49] Kessler RC, Sonnega A, Bromet E, Hughes M, Nelson CB (1995). Posttraumatic stress disorder in the national comorbidity survey. *Archives of General Psychiatry*.

[50] Felitti VJ, Anda RF, Nordenberg D (1998). Relationship of childhood abuse and household dysfunction to many of the leading causes of death in adults: the Adverse Childhood Experiences (ACE) study. *American Journal of Preventive Medicine*.

[51] Dube SR, Anda RF, Felitti VJ, Chapman DP, Williamson DF, Giles WH (2001). Childhood abuse, household dysfunction, and the risk of attempted suicide throughout the life span: findings from the adverse childhood experiences study. *Journal of the American Medical Association*.

[52] Anda RF, Brown DW, Felitti VJ, Dube SR, Giles WH (2008). Adverse childhood experiences and prescription drug use in a cohort study of adult HMO patients. *BMC Public Health*.

[53] Dube SR, Anda RF, Felitti VJ, Edwards VJ, Croft JB (2002). Adverse childhood experiences and personal alcohol abuse as an adult. *Addictive Behaviors*.

[54] Anda RF, Whitfield CL, Felitti VJ (2002). Adverse childhood experiences, alcoholic parents, and later risk of alcoholism and depression. *Psychiatric Services*.

[55] Danese A, Tan M (2013). Childhood maltreatment and obesity: systematic review and meta-analysis. *Molecular Psychiatry*.

[56] Duke NN, Pettingell SL, McMorris BJ, Borowsky IW (2010). Adolescent violence perpetration: associations with multiple types of adverse childhood experiences. *Pediatrics*.

[57] Flaherty EG, Schwartz K, Jones RD, Sege RD (2013). Child abuse physicians: coping with challenges. *Evaluation & the Health Professions*.

[58] Strachan T, Read A (2010). Genes in pedigrees and populations. *Human Molecular Genetics*.

[59] Mehta D, Gonik M, Klengel T (2011). Using polymorphisms in FKBP5 to define biologically distinct subtypes of posttraumatic stress disorder: evidence from endocrine and gene expression studies. *Archives of General Psychiatry*.

[60] Aguilar-Salinas CA, Canizales-Quinteros S, Rojas-Martfnez R (2009). Hypoalphalipoproteinemia in populations of Native American ancestry: an opportunity to assess the interaction of genes and the environment. *Current Opinion in Lipidology*.

[61] Mehta D, Klengel T, Conneely KN (2013). Childhood maltreatment is associated with distinct genomic and epigenetic profiles in posttraumatic stress disorder. *Proceedings of the National Academy of Sciences of the United States of America*.

[62] Timmermans W, Xiong H, Hoogenraad CC, Krugers HJ (2013). Stress and excitatory synapses: from health to disease. *Neuroscience*.

[63] Ceccatelli S, Tamm C, Zhang Q, Chen M (2007). Mechanisms and modulation of neural cell damage induced by oxidative stress. *Physiology and Behavior*.

[64] Duman RS (2009). Neuronal damage and protection in the pathophysiology and treatment of psychiatric illness: stress and depression. *Dialogues in Clinical Neuroscience*.

[65] Epel ES (2009). Psychological and metabolic stress: a recipe for accelerated cellular aging?. *Hormones*.

[66] Kalil GZ, Haynes WG (2012). Sympathetic nervous system in obesity-related hypertension: mechanisms and clinical implications. *Hypertension Research*.

[67] Kim-Cohen J, Moffitt TE, Caspi A, Taylor A (2004). Genetic and environmental processes in young children’s resilience and vulnerability to socioeconomic deprivation. *Child Development*.

[68] McGowan PO, Sasaki A, D’Alessio AC (2009). Epigenetic regulation of the glucocorticoid receptor in human brain associates with childhood abuse. *Nature Neuroscience*.

[69] Roth TL, Sweatt JD (2011). Epigenetic marking of the BDNF gene by early-life adverse experiences. *Hormones and Behavior*.

[70] Hunter RG, McEwen BS (2013). Stress and anxiety across the lifespan: structural plasticity and epigenetic regulation. *Epigenomics*.

[71] Weaver ICG, Cervoni N, Champagne FA (2004). Epigenetic programming by maternal behavior. *Nature Neuroscience*.

[72] Liu D, Diorio J, Tannenbaum B (1997). Maternal care, hippocampal glucocorticoid receptors, and hypothalamic-pituitary-adrenal responses to stress. *Science*.

[73] Sanchez MM (2006). The impact of early adverse care on HPA axis development: nonhuman primate models. *Hormones and Behavior*.

[74] Fumagalli F, Molteni R, Racagni G, Riva MA (2007). Stress during development: impact on neuroplasticity and relevance to psychopathology. *Progress in Neurobiology*.

[75] Koshibu K, Gräff J, Mansuy IM (2011). Nuclear protein phosphatase-1: an epigenetic regulator of fear memory and amygdala long-term potentiation. *Neuroscience*.

[76] Roth TL, Zoladz PR, Sweatt JD, Diamond DM (2011). Epigenetic modification of hippocampal Bdnf DNA in adult rats in an animal model of post-traumatic stress disorder. *Journal of Psychiatric Research*.

[77] Labonté B (2012). Genome-wide epigenetic regulation by early-life trauma. *Archives of General Psychiatry*.

[78] McGowan PO, Sasaki A, D’Alessio AC (2009). Epigenetic regulation of the glucocorticoid receptor in human brain associates with childhood abuse. *Nature Neuroscience*.

[79] Ohgane J, Yagi S, Shiota K (2008). Epigenetics: the DNA methylation profile of tissue-dependent and differentially methylated regions in cells. *Placenta*.

[80] Nagase H, Ghosh S (2008). Epigenetics: differential DNA methylation in mammalian somatic tissues. *FEBS Journal*.

[81] Klengel T, Mehta D, Anacker C (2013). Allele-specific FKBP5 DNA demethylation mediates gene-childhood trauma interactions. *Nature Neuroscience*.

[82] Tyrka AR, Price LH, Marsit C, Walters OC, Carpenter LL (2012). Childhood adversity and epigenetic modulation of the leukocyte glucocorticoid receptor: preliminary findings in healthy adults. *PLoS ONE*.

[83] Oberlander TF, Weinberg J, Papsdorf M, Grunau R, Misri S, Devlin AM (2008). Prenatal exposure to maternal depression, neonatal methylation of human glucocorticoid receptor gene (NR3C1) and infant cortisol stress responses. *Epigenetics*.

[84] Yang BZ, Zhang H, Ge W (2013). Child abuse and epigenetic mechanisms of disease risk. *American Journal of Preventive Medicine*.

[85] Essex MJ, Boyce WT, Hertzman C (2013). Epigenetic vestiges of early developmental adversity: childhood stress exposure and DNA methylation in adolescence. *Child Development*.

[86] Borghol N, Suderman M, Mcardle W (2012). Associations with early-life socio-economic position in adult DNA methylation. *International Journal of Epidemiology*.

[87] Beach SRH, Brody GH, Todorov AA, Gunter TD, Philibert RA (2010). Methylation at SLC6A4 is linked to family history of child abuse: an examination of the Iowa adoptee sample. *American Journal of Medical Genetics B*.

[88] Beach SRH, Brody GH, Todorov AA, Gunter TD, Philibert RA (2011). Methylation at 5HTT mediates the impact of child sex abuse on women’s antisocial behavior: an examination of the iowa adoptee sample. *Psychosomatic Medicine*.

[89] Vijayendran M, Beach SR, Plume JM, Brody GH, Philibert RA (2012). Effects of genotype and child abuse on DNA methylation and gene expression at the serotonin transporter. *Front Psychiatry*.

[90] Uddin M, Aiello AE, Wildman DE (2010). Epigenetic and immune function profiles associated with posttraumatic stress disorder. *Proceedings of the National Academy of Sciences of the United States of America*.

[91] Rusiecki JA, Chen L, Srikantan V (2012). DNA methylation in repetitive elements and post-traumatic stress disorder: a case-control study of US military service members. *Epigenomics*.

[92] Koenen KC, Uddin M, Chang S-C (2011). SLC6A4 methylation modifies the effect of the number of traumatic events on risk for posttraumatic stress disorder. *Depression and Anxiety*.

[93] Uddin M, Galea S, Chang S-C (2011). Gene expression and methylation signatures of MAN2C1 are associated with PTSD. *Disease Markers*.

[94] Sabunciyan S, Aryee MJ, Irizarry RA (2012). Genome-wide DNA methylation scan in major depressive disorder. *PLoS ONE*.

[95] Zill P, Baghai TC, Schüle C (2012). DNA methylation analysis of the Angiotensin Converting Enzyme (ACE) gene in major depression. *PLoS ONE*.

[96] Fuchikami M, Morinobu S, Segawa M (2011). DNA methylation profiles of the Brain-Derived Neurotrophic Factor (BDNF) gene as a potent diagnostic biomarker in major depression. *PLoS ONE*.

[97] Rotter A, Asemann R, Decker A, Kornhuber J, Biermann T (2011). Orexin expression and promoter-methylation in peripheral blood of patients suffering from major depressive disorder. *Journal of Affective Disorders*.

[98] Poulter MO, Du L, Weaver ICG (2008). GABAA receptor promoter hypermethylation in suicide brain: implications for the involvement of epigenetic processes. *Biological Psychiatry*.

[99] Doehring A, Oertel BG, Sittl R, Lötsch L (2013). Chronic opioid use is associated with increased DNA methylation correlating with increased clinical pain. *Pain*.

[100] Chorbov VM, Todorov AA, Lynskey MT, Cicero TJ (2011). Elevated levels of DNA methylation at the OPRM1 promoter in blood and sperm from male opioid addicts. *Journal of Opioid Management*.

[101] Nielsen DA, Hamon S, Yuferov V (2010). Ethnic diversity of DNA methylation in the OPRM1 promoter region in lymphocytes of heroin addicts. *Human Genetics*.

[102] Nielsen DA, Yuferov V, Hamon S (2009). Increased OPRM1 DNA methylation in lymphocytes of methadone-maintained former heroin addicts. *Neuropsychopharmacology*.

[103] Zhang H, Herman AI, Kranzler HR, Anton RF, Simen AA, Gelernter J (2012). Hypermethylation of OPRM1 promoter region in European Americans with alcohol dependence. *Journal of Human Genetics*.

[104] Manzardo AM, Henkhaus RS, Butler MG (2012). Global DNA promoter methylation in frontal cortex of alcoholics and controls. *Gene*.

[105] Muschler MAN, Hillemacher T, Kraus C, Kornhuber J, Bleich S, Frieling H (2010). DNA methylation of the POMC gene promoter is associated with craving in alcohol dependence. *Journal of Neural Transmission*.

[106] Kongkaew C, Noyce PR, Ashcroft DM (2008). Hospital admissions associated with adverse drug reactions: a systematic review of prospective observational studies. *Annals of Pharmacotherapy*.

[107] Bleich S, Lenz B, Ziegenbein M (2006). Epigenetic DNA hypermethylation of the HERP gene promoter induces down-regulation of its mRNA expression in patients with alcohol dependence. *Alcoholism*.

[108] Bönsch D, Lenz B, Kornhuber J, Bleich S (2005). DNA hypermethylation of the alpha synuclein promoter in patients with alcoholism. *NeuroReport*.

[109] Philibert RA, Gunter TD, Beach SRH, Brody GH, Madan A (2008). Rapid publication: MAOA methylation is associated with nicotine and alcohol dependence in women. *American Journal of Medical Genetics B*.

